# Supplementary material for: Modeling glioblastoma heterogeneity as a dynamic network of cell states
Source: Mol Syst Biol. 2021 Sep 16;17(9):e10105. doi: 10.15252/msb.202010105 (PMC8444284; doi:10.15252/msb.202010105)
Supplement: Supplementary file 6 — Source Data for Figure 5 [file MSB-17-e10105-s004.zip › Figure5A_sourcedata/GSEA_3017/nosigdb_stateD.GseaPreranked.1622795010397/RICHARDS_NOWAKOWSKI_SCIENCE_2017_NIN5_UPREG.html]

Details for gene set RICHARDS\_NOWAKOWSKI\_SCIENCE\_2017\_NIN5\_UPREG[GSEA]

|  || Dataset | state23017 |
| Phenotype | NoPhenotypeAvailable |
| Upregulated in class | na\_pos |
| GeneSet | RICHARDS\_NOWAKOWSKI\_SCIENCE\_2017\_NIN5\_UPREG |
| Enrichment Score (ES) | 0.12328199 |
| Normalized Enrichment Score (NES) | 1.0491484 |
| Nominal p-value | 0.33333334 |
| FDR q-value | 0.51090884 |
| FWER p-Value | 0.97 |
Table: GSEA Results Summary

  

Fig 1: Enrichment plot: RICHARDS\_NOWAKOWSKI\_SCIENCE\_2017\_NIN5\_UPREG      
 Profile of the Running ES Score & Positions of GeneSet Members on the Rank Ordered List

  

| PROBE | GENE SYMBOL | GENE\_TITLE | RANK IN GENE LIST | RANK METRIC SCORE | RUNNING ES | CORE ENRICHMENT || 1 | SOX11 |  |  | 6 | 0.578 | 0.0311 | Yes |
| 2 | ARL4C |  |  | 24 | 0.422 | 0.0439 | Yes |
| 3 | EIF5A |  |  | 90 | 0.301 | 0.0115 | Yes |
| 4 | SOX4 |  |  | 91 | 0.298 | 0.0300 | Yes |
| 5 | MARCKS |  |  | 96 | 0.295 | 0.0452 | Yes |
| 6 | IER2 |  |  | 101 | 0.290 | 0.0600 | Yes |
| 7 | CHD7 |  |  | 116 | 0.278 | 0.0662 | Yes |
| 8 | TUBA1A |  |  | 148 | 0.259 | 0.0580 | Yes |
| 9 | PLXNA4 |  |  | 182 | -0.252 | 0.0477 | Yes |
| 10 | RBM23 |  |  | 231 | -0.257 | 0.0259 | Yes |
| 11 | ZEB2 |  |  | 317 | -0.265 | -0.0244 | Yes |
| 12 | ARMC1 |  |  | 326 | -0.266 | -0.0142 | Yes |
| 13 | PELI2 |  |  | 336 | -0.267 | -0.0047 | Yes |
| 14 | MYO9A |  |  | 356 | -0.268 | -0.0030 | Yes |
| 15 | ATXN2 |  |  | 389 | -0.272 | -0.0113 | Yes |
| 16 | CARHSP1 |  |  | 405 | -0.274 | -0.0061 | Yes |
| 17 | TUBB |  |  | 410 | -0.274 | 0.0078 | Yes |
| 18 | CSE1L |  |  | 421 | -0.276 | 0.0171 | Yes |
| 19 | BNIP3L |  |  | 431 | -0.277 | 0.0272 | Yes |
| 20 | DLX1 |  |  | 490 | -0.284 | -0.0008 | Yes |
| 21 | DDR1 |  |  | 491 | -0.284 | 0.0168 | Yes |
| 22 | KCND3 |  |  | 500 | -0.285 | 0.0282 | Yes |
| 23 | NUDT3 |  |  | 504 | -0.285 | 0.0435 | Yes |
| 24 | ERC1 |  |  | 507 | -0.286 | 0.0597 | Yes |
| 25 | USP9Y |  |  | 516 | -0.287 | 0.0712 | Yes |
| 26 | ABCA1 |  |  | 563 | -0.293 | 0.0532 | Yes |
| 27 | NEDD4L |  |  | 595 | -0.299 | 0.0474 | Yes |
| 28 | CCDC93 |  |  | 610 | -0.303 | 0.0552 | Yes |
| 29 | ACTR10 |  |  | 615 | -0.303 | 0.0708 | Yes |
| 30 | ZNF83 |  |  | 630 | -0.307 | 0.0789 | Yes |
| 31 | ACADM |  |  | 636 | -0.308 | 0.0940 | Yes |
| 32 | ATP9A |  |  | 692 | -0.318 | 0.0705 | Yes |
| 33 | ST8SIA5 |  |  | 737 | -0.327 | 0.0563 | Yes |
| 34 | PPP1R12A |  |  | 741 | -0.329 | 0.0743 | Yes |
| 35 | TMSB4X |  |  | 742 | -0.329 | 0.0947 | Yes |
| 36 | PTPRA |  |  | 772 | -0.337 | 0.0928 | Yes |
| 37 | RND3 |  |  | 853 | -0.358 | 0.0522 | Yes |
| 38 | PRKDC |  |  | 871 | -0.363 | 0.0614 | Yes |
| 39 | TMEM106B |  |  | 886 | -0.367 | 0.0732 | Yes |
| 40 | MEG3 |  |  | 975 | -0.404 | 0.0291 | Yes |
| 41 | SUGP2 |  |  | 982 | -0.407 | 0.0497 | Yes |
| 42 | DPYSL5 |  |  | 983 | -0.408 | 0.0750 | Yes |
| 43 | DCLK2 |  |  | 986 | -0.408 | 0.0987 | Yes |
| 44 | KIFAP3 |  |  | 988 | -0.409 | 0.1233 | Yes |
| 45 | NCAM1 |  |  | 1049 | -0.445 | 0.1038 | No |
| 46 | FTH1 |  |  | 1152 | -0.514 | 0.0555 | No |
| 47 | GRIA1 |  |  | 1170 | -0.538 | 0.0755 | No |
| 48 | GRIA4 |  |  | 1238 | -0.669 | 0.0644 | No |
Table: GSEA details [plain text format]

  

Fig 2: RICHARDS\_NOWAKOWSKI\_SCIENCE\_2017\_NIN5\_UPREG: Random ES distribution      
 Gene set null distribution of ES for **RICHARDS\_NOWAKOWSKI\_SCIENCE\_2017\_NIN5\_UPREG**
